# Comparing longitudinal patient-reported outcome measures between Swedish patients with recent-onset systemic lupus erythematosus and early rheumatoid arthritis

**DOI:** 10.1007/s10067-021-05982-3

**Published:** 2021-11-27

**Authors:** Rebecca Heijke, Mathilda Björk, Ingrid Thyberg, Alf Kastbom, Laura McDonald, Christopher Sjöwall

**Affiliations:** 1 Division of Inflammation and Infection, Department of Biomedical and Clinical Sciences, Linköping University, SE-581 85 Linköping, Sweden; 2 Department of Health, Medicine and Caring Sciences, Linköping University, Norrköping, Sweden; 3 Real-World Research, Bristol Myers Squibb, Uxbridge Business Park, Sanderson Road, Uxbridge, UK

**Keywords:** Patient-reported outcome measures, Quality of life, Recent onset of disease, Rheumatoid arthritis, Systemic lupus erythematosus

## Abstract

The onset of rheumatic disease affects each patient differently and may impact quality of life with progression. We investigated the relationship between patient-reported outcome measure (PROM) scores and organ damage in patients with recent-onset systemic lupus erythematosus (SLE) and those with early rheumatoid arthritis (RA). Patients with recent-onset SLE without prior organ damage from the *Clinical Lupus Register in Northeastern Gothia* and patients with early RA from the observational *2nd Timely Interventions in Early RA *study, Sweden, were included. Systemic Lupus International Collaborating Clinics/American College of Rheumatology damage index (SDI) was used to assess organ damage. PROM (visual analog scale [VAS]: pain, fatigue, well-being, Health Assessment Questionnaire, and EQ-5D-3L) scores were captured at months 0, 6, 12, 24, 36, 48, and 60 after diagnosis. Statistical tests included Pearson correlation coefficients and *t-*tests. Forty-one patients with recent-onset SLE and 522 with early RA were included. Numerical differences were seen in age and sex. PROMs were worse for patients with RA versus SLE but improved by month 6 following diagnosis, while SLE PROMs remained stable. The incidence of organ damage in SLE was 13.6 per 100 patient-years. SDI significantly correlated with EQ-5D-3L (− 0.48, *P* = 0.003), VAS fatigue (0.44, *P* = 0.009), and well-being (0.41, *P* = 0.01) at month 24. As illustrated, the complexity of disease burden in patients with SLE is clear and may result from disease-related multiorgan system effects and slower symptom resolution compared with RA. This underscores the need for improved multiprofessional interventions to manage all aspects of SLE.

**Table:** 

**Key Points** • * We observed an evident discrepancy in patient-reported outcome measures (PROMs) between patients with recent-onset SLE and early RA*. • *Despite differences in PROMs between patients with recent-onset SLE and early RA, both groups had prominent self-reported disability during the study period*. • * PROM scores for patients with RA were generally worse than those with SLE but improved by month 6, whereas PROM scores for patients with SLE remained stable over time*. • *Our findings underline the need of new therapeutic options and interventions for SLE disease management, including pharmacologic and multiprofessional aspects*.

**Key Points**

• * We observed an evident discrepancy in patient-reported outcome measures (PROMs) between patients with recent-onset SLE and early RA*.

• *Despite differences in PROMs between patients with recent-onset SLE and early RA, both groups had prominent self-reported disability during the study period*.

• * PROM scores for patients with RA were generally worse than those with SLE but improved by month 6, whereas PROM scores for patients with SLE remained stable over time*.

• *Our findings underline the need of new therapeutic options and interventions for SLE disease management, including pharmacologic and multiprofessional aspects*.

## Introduction

Systemic lupus erythematosus (SLE) is a chronic disease with multiorgan involvement that may result in irreversible organ damage [1, 2]. Despite improved treatments and survival, SLE significantly affects quality of life (QoL) and general health [1]. The worldwide incidence and prevalence of SLE vary significantly between populations, with most patients who are women of childbearing age [3]. Prevalence estimates in Sweden range from 39 to 85 cases per 100,000 persons (0.04–0.10%) [3]. A German study showed that healthcare resource utilization and costs for incident SLE cases were higher than prevalent cases [4], emphasizing the need for improved healthcare interventions as the patient experience changes over time. Apart from direct costs, Swedish data indicate that indirect costs are substantial in established SLE [5].

Acquired organ damage in SLE is a result of disease progression, treatment side effects, comorbidities, or unrelated events and accumulates gradually at different rates across organ systems [6]. In SLE, damage accrual is an important outcome measure and is associated with reduced QoL [2]. The Systemic Lupus International Collaborating Clinics/American College of Rheumatology Damage Index (SLICC/ACR DI; SDI), a validated instrument, is widely used for this purpose [6, 7].

Our previous studies show relationships between activity limitations, disease activity, and QoL in patients with SLE [2, 8]. One study found that the presence of activity limitations detected by Health Assessment Questionnaire (HAQ) scores were significantly associated with QoL (EQ-5D-3L) and acquired organ damage (SDI) [2]. Another found significant correlation between disease activity with pain and well-being [8]. Validated instruments including the HAQ, EQ-5D-3L, and visual analog scale (VAS, pain, fatigue, well-being) are frequently used to measure patient-reported outcomes in rheumatic disease [2, 8–11].

Although associations between traditional measures of damage accrual and SLE disease progression are well established [6], the relationship between SDI and patient-reported outcome measures (PROMs) is unclear [1, 2]. Improvements in disease activity, functional disability, and health-related QoL are often seen within 1 year after diagnosis among patients with RA, but comparable studies in SLE are scarce [12]. Combining conventional physician assessments with PROMs could enhance communication and shared decision-making between patients with SLE and their healthcare providers [1].

The primary aim of this study was to compare changes in PROM scores among patients with recent-onset SLE and early RA for 60 months. Secondly, we aimed to reveal potential correlations between organ damage and PROMs in SLE.

## Materials and methods

### Data source, patients, and study design

This was a 60-month observational study of 41 recent-onset cases of SLE and 522 cases of early RA. Patients with SLE were included in the *Clinical Lupus Register in Northeastern Gothia* (KLURING), Sweden, at the University Hospital in Linköping. The KLURING database was previously described in detail [2].

Eligible patients with SLE were ≥ 18 years of age, newly diagnosed with SLE, fulfilled ≥ 4 of the 1982 ACR (ACR-82) [13] and/or the 2012 SLICC classification criteria [14], and had no prior organ damage. Data were retrieved for eligible patients recruited between March 2010 and October 2015 and seen by a rheumatologist at 0 (index date), 6, 12, 24, 36, 48, and 60 months after diagnosis (± 3 months per visit). The schedule was based on international recommendations for management and follow-up of patients with early SLE and RA [15, 16].

Patients with RA from the observational *2nd Timely Interventions in Early RA* study (TIRA-2) were included as a comparator group. TIRA-2 enrolled patients with early RA from January 2006 through August 2009 (detailed previously) [17]. Patients in SLE and RA groups had symptoms for < 1 year before diagnosis, were treated according to Swedish national guidelines, and followed prospectively. Patients with RA were selected as a comparator group given the similarities with SLE regarding disease progression, joint involvement, disability, and manifestations [10, 11, 18]. Apart from musculoskeletal involvement, constitutional, neuropsychiatric, pulmonary, and cardiac manifestations are frequent in RA [18, 19].

All patients gave oral and/or written informed consent [2, 17]. The study met ethical standards for human and animal rights, and protocols were approved by the regional ethical review board in Linköping (Decision No. M168–05 and M75–08/2008).

### Study assessments

We used clinical measures and PROMs at months 0, 6, 12, 24, 36, 48, and 60. Organ damage was assessed using the SDI encompassing 12 organ systems [7]. For SLE, the incidence of acquired organ damage, defined by an SDI [6, 7] of > 0 was assessed for the study period and each calendar year (2010–2017). Overall incidence was stratified by organ system. Incidence rates were calculated as the number of incident cases of organ damage during the study period over person-time at risk (time patient remained in database without organ damage). PROMs included were as follows: the Swedish versions of the HAQ to assess functional disability (0 = no difficulty; 1 = unable to do), the EQ-5D-3L to assess general health (1 = perfect health; 0 = dead), and the VAS (0–100 mm; 0 = no impairment; 100 = complete impairment) to assess pain, fatigue, and well-being [20–22].

To assess clinically important differences across outcomes, we used published values: for SDI, standard error of measure (minimum clinically important difference [MCID] = 2) [7]; for HAQ, 0.22 (range: 0.07–0.87) [23]; for EQ-5D-3L, 0.29 (range: 0.03–0.54) [24]; and for VAS, by age-group, ranged from 7 (aged 30–49) to 37 units (aged > 65) [25].

All PROMs (VAS [26, 27]; EQ-5D-3L [28, 29]; HAQ [20, 30]) were validated in respective patient populations.

### Statistical analyses

Descriptive statistics were used to assess baseline characteristics and PROMs at registry entry and follow-up visits. Cross-sectional correlations between organ damage and PROMs were examined using Pearson correlation coefficients and *P* values. Comparisons of baseline characteristics and PROM scores for patients with recent-onset SLE versus early RA were assessed using *t*-tests (Mann-Whitney *U* or Fisher’s exact tests). All measures were assessed at baseline and at follow-up visits through month 60.

## Results

### Patients

A total of 41 patients with recent-onset SLE and 522 patients with early RA were included (Table 1) [8, 13, 14, 31]. Detailed patient characteristics were reported previously [8, 17]. Among patients with SLE, median age was 39 years, 80.5% were female, and 36.6% had lupus nephritis [8]. Complete 60-month data were available for 20 patients (48.8%). For patients with RA, median age was 60 years, and 67.4% were female. (Table 1).

**Table 1:** Patient characteristics at study entry

Characteristic	SLE (*n* = 41)	RA (*n* = 522)
Age, median (range), years	39.0 (18–77)	60.0 (51–68)
Female	33 (80.5)	352 (67.4)
White	35 (85.4)	N.C.
Cases fulfilled meeting the 1982 ACR SLE criteria [13]	36 (87.8)	N.A.
Number of 1982 ACR SLE criteria fulfilled, mean (range)	4.7 (3–9)	N.A.
Cases meeting the 2012 SLICC criteria [14]	41 (100)	N.A.
Cases meeting the 1987 ACR RA criteria [31]	N.A.	439 (84.1)

Data are *n* (%) unless otherwise indicated

The SLE study population has previously been detailed [8]

*ACR* American College of Rheumatology, *N.A.* not applicable, *N.C.* not collected, *SLICC* Systemic Lupus International Collaborating Clinics, *RA* rheumatoid arthritis, *SLE* systemic lupus erythematosus

### Incidence of organ damage in SLE

**Fig. 1:**
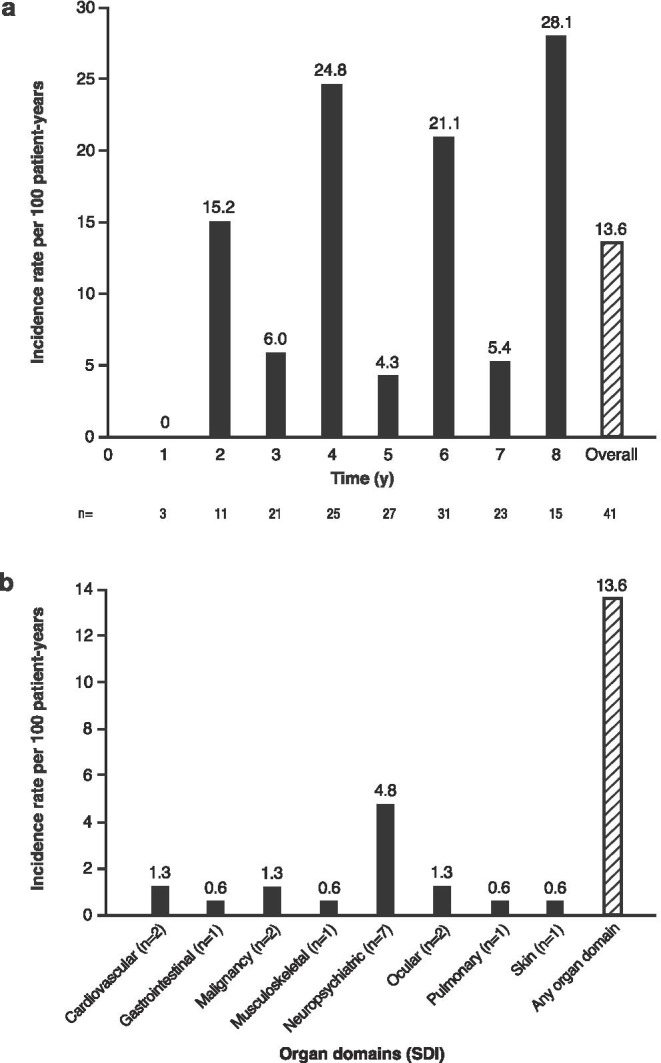
Incidence of organ damage in recent-onset SLE: **a** incidence rate of organ damage by calendar year and overall, a subgroup of patients was followed for a longer time period than 60 months; **b** incidence rate of organ damage by organ domain and overall. Bars represent the incidence rate per 100 patient-years. In (**a**), *n* = indicates the number of patients active in the calendar year; the sum across years will not match the overall count of 41. SDI, SLICC/ACR damage index; SLE, systemic lupus erythematosus; SLICC/ACR, Systemic Lupus International Collaborating Clinics/American College of Rheumatology

The incidence of organ damage from 2010 to 2017 was 13.6 per 100 patient-years, and no clear relationship between incidence of organ damage and year after registry entry was observed (Fig. 1a). Of the organ domains examined, the neuropsychiatric domain showed the highest incidence of organ damage (SDI: 4.8 per 100 patient-years) (Fig. 1b).

### Correlations between clinical outcomes and PROMs in SLE

After SLE diagnosis, a numerical increase in SDI was observed during the 60-month period; however, PROM scores remained stable. Significant correlations were observed between the SDI and EQ-5D-3L (−0 .48, *P* = 0.003), VAS fatigue (0.44, *P* = 0.009), and well-being (0.41, *P* = 0.01) at month 24 (Table 2). No other correlations were observed. Completers at 60-months (*n* = 20) were compared with non-completers (*n* = 21) for all PROMs. At month 0, the VAS fatigue score was lower in completers (*P* < 0.0001). At month 6, the VAS pain score was higher among non-completers (*P* = 0.04), whereas at month 36, pain was significantly lower (*P* = 0.02).

**Table 2:** Pearson correlations between SLICC/ACR Damage Index (SDI) and PROMs for patients with SLE

			SDI and EQ-5D-3L		SDI and VAS pain		SDI and VAS fatigue		SDI and VAS well-being	
Mo. since diagnosis	n	SDI, mean ± SD	PCC	*P* value	PCC	*P* value	PCC	*P* value	PCC	*P* value
0	41	0.0	N.A.	N.A.	N.A.	N.A.	N.A.	N.A.	–	–
6	31	0.19 ± 0.48	0.11	N.S.	– 0.02	N.S.	− 0.08	N.S.	0.12	N.S.
12	37	0.24 ± 0.49	− 0.29	N.S.	0.20	N.S.	0.31	N.S.	− 0.03	N.S.
24	36	0.33 ± 0.53	− 0.48	0.003	0.32	N.S.	0.44	0.009	0.41	0.01
36	30	0.43 ± 0.63	− 0.32	N.S.	– 0.16	N.S.	0.00	N.S.	− 0.08	N.S.
48	23	0.57 ± 0.73	− 0.26	N.S.	– 0.01	N.S.	0.16	N.S.	0.07	N.S.
60	20	0.45 ± 0.60	0.04	N.S.	– 0.14	N.S.	0.03	N.S.	0.00	N.S.

*N.A.* not applicable, *N.S.* not significant, *PCC* Pearson correlation coefficient, *PROMs* patient-reported outcome measures, *SD* standard deviation, *SDI* Systemic Lupus International Collaborating Clinics/American College of Rheumatology Damage Index, *SLE* systemic lupus erythematosus, *VAS*, visual analog scale

### Comparison of PROMs in SLE versus RA

**Fig. 2:**
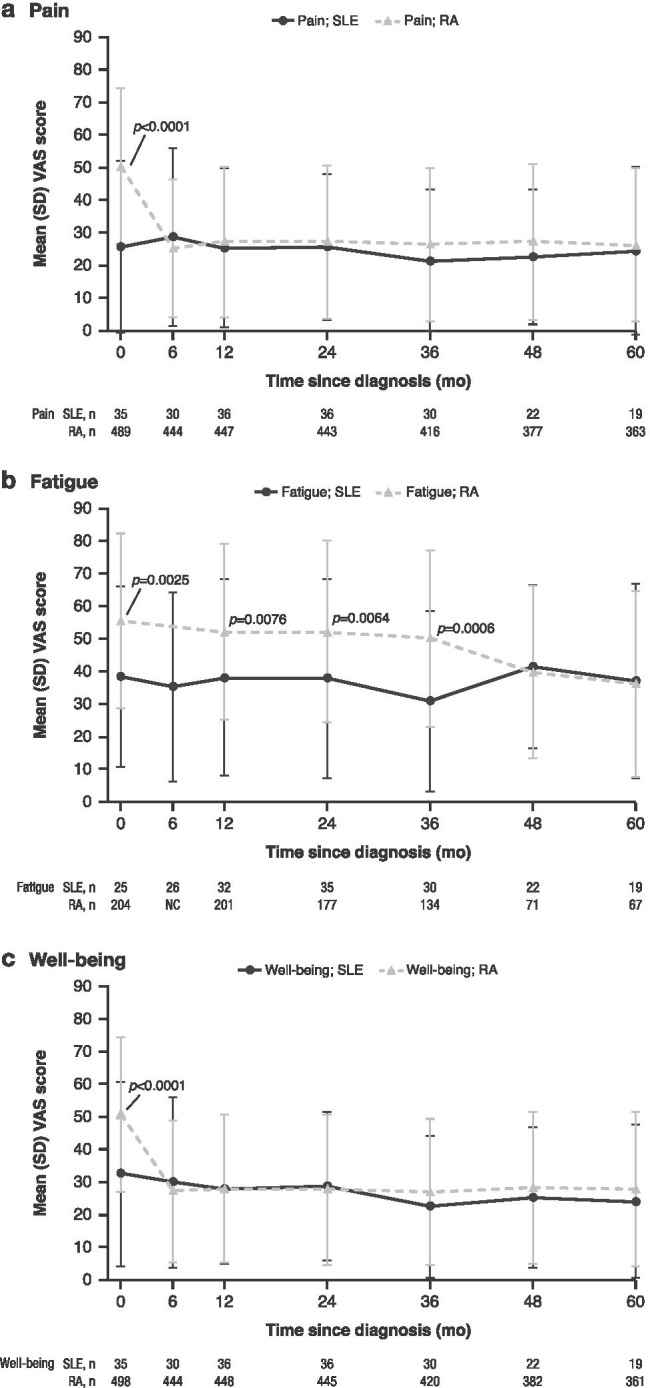
Mean PROM scores over time in patients with SLE and RA for **a** VAS pain, **b** VAS fatigue, and **c** VAS well-being. Data represent mean ± SD at each time point; *n* = number of observations. VAS pain, fatigue, and well-being (range: 0 to 100 mm): higher scores indicate greater severity. VAS fatigue data were not collected at month 6 for RA. Patients with missing data were not included in the mean (SD) calculations for that time point. Baseline VAS pain (25.69 vs 50.48) *P* < 0.0001 (Fig. 2a); baseline VAS fatigue (38.32 vs 55.73) *P* = 0.0025; 12 months (38.10 vs 52.18) *P* = 0.0076*;* 24 months (37.86 vs 52.29) *P* = 0.0064; 36 months (30.90 vs 50.19), *P =* 0.0006 (Fig. 2b); baseline VAS well-being (32.60 vs 50.87) *P* < 0.0001 (Fig. 2c); *P* values for all VAS domains indicate significantly higher scores in patients with RA versus SLE at baseline. *P* values for VAS fatigue indicate significantly higher scores for patients with RA versus SLE at 12, 24, and 36 months. NC, not collected; RA, rheumatoid arthritis; SD, standard deviation; SLE, systemic lupus erythematosus; VAS, visual analog scale

**Fig. 3:**
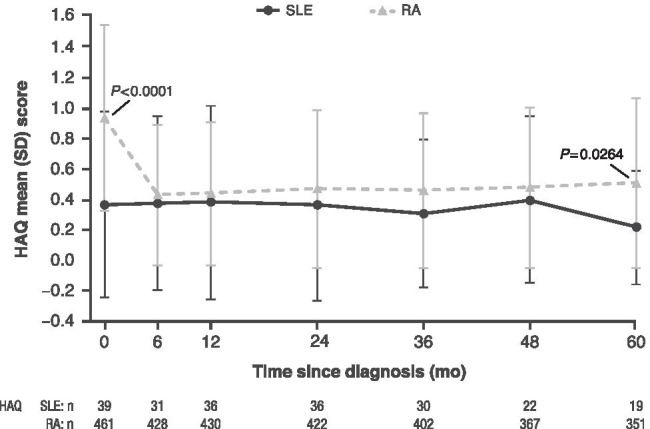
Mean PROM scores over time in patients with SLE and RA for HAQ. Baseline HAQ (0.36 vs 0.94) *P* < 0.0001; 60 months (0.37 vs 0.51) *P* = 0.0264. *P* values for HAQ indicate significantly higher scores in RA versus SLE patients at baseline and 60 months. HAQ, Health Assessment Questionnaire; PROM, patient-reported outcome measures; RA, rheumatoid arthritis; SLE, systemic lupus erythematosus

**Fig. 4:**
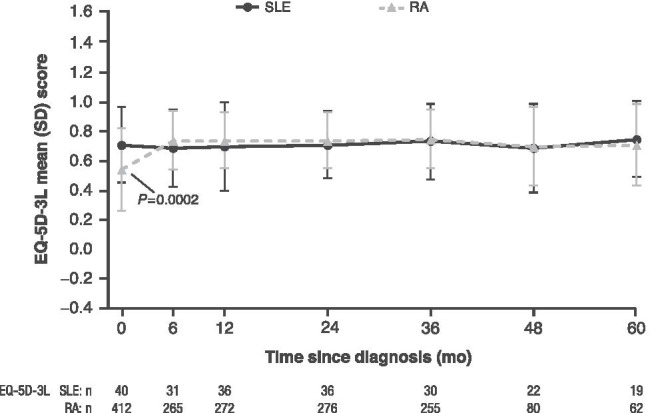
Mean PROM scores over time in patients with SLE and RA for EQ-5D-3L. Baseline EQ-5D-3L (0.71 vs 0.54) *P* = 0.0002. Consistent with VAS and HAQ scores, QoL was significantly lower for patients with RA than SLE. PROM, patient-reported outcome measures; QoL, quality of life; RA, rheumatoid arthritis; SLE, systemic lupus erythematosus; VAS, visual analog scale

Baseline PROM scores were generally worse for patients with RA versus SLE, with significant differences for VAS pain (*P <* 0.0001), well-being (*P <* 0.0001), fatigue (*P<* 0.05) (Fig. 2), and functional disability (HAQ) (*P <* 0.0001) (Fig. 3). Unlike for patients with SLE, improvement in PROM scores was seen by month 6 for RA. Scores remained largely unchanged across cohorts but were still affected between months 6 and 60. Consistent with this result, patients with RA had a significantly lower baseline QoL versus SLE (EQ-5D-3L: *P* = 0.0002) (Fig. 4). Additionally, VAS fatigue scores for patients with RA were significantly higher at months 12 (*P* = 0.0076), 24 (*P* = 0.0064), and 36 (*P* = 0.0006) (Fig. 2). HAQ scores were significantly higher at month 60 for patients with RA versus SLE (*P* = 0.0264) (Fig. 3).

## Discussion

Using longitudinal data from a well-characterized Swedish cohort, we show that patients with SLE had lower, but still affected, PROM scores compared with patients with RA at baseline. In contrast to RA, there was no significant or clinically relevant improvement in PROM scores for SLE over time. No significant correlations were observed between organ damage and PROM scores except for a correlation between EQ-5D-3L score and fatigue at month 24. Patients with RA had significantly worse PROM scores at baseline with clinically significant improvement experienced by month 6. Given PROMs in recent-onset SLE remained stable but affected relative to healthy norms through 60 months, indicates a greater unmet need for patients with SLE than for patients with RA.

Symptoms and complications of SLE can substantially affect patients’ QoL. Herein, patients with RA and SLE reported worse QoL than found in the general Swedish population [32]. We previously demonstrated associations between HAQ scores and QoL in patients with SLE; greater limitations in activity level were associated with lower QoL [2].

The clinical and QoL implications of SLE underscore the importance of obtaining current estimates of the incidence of organ damage in SLE and clarifying the relationship between clinical outcomes and QoL. Both cohorts show affected PROM scores during the first 5 years after disease onset. In contrast to improvement in PROMs observed by month 6 among patients with RA, the lack of improvement among patients with SLE may be explained by the disease-related effects across organ systems, which may take longer to resolve than the symptoms of RA. Compared with RA, patients with SLE appear to be underserved concerning clinical evaluation for activity limitations and self-reported health. These results underscore the need for more effective measures and interventions for SLE disease management, including pharmacologic and multiprofessional healthcare services.

A major strength of this study is the Swedish healthcare system (public, tax-funded, and universal access). This significantly reduces the risk of selection bias and ensures high case coverage, especially at a single tertiary referral center that offers highly specialized multiprofessional services from skilled practitioners in rheumatologic care [17]. Furthermore, by including only incident cases, PROMs data were unbiased from previous organ damage. The KLURING registry is a rich data source that provides detailed clinical characteristics and evaluations of multiple outcome measures over time. Using a large control group (RA) also places results into a broader context. The primary limitation is the small sample size of the SLE cohort. This may restrict the ability to evaluate confounding factors that may lead to imprecise estimates. Significant correlations between SDI and EQ-5D-3L and fatigue at only month 24 should also be interpreted with caution given the small sample size [8]. Lastly, because most patients were White, results may not be generalizable across races and ethnicities.

In a companion study with the same 41 Swedish patients with recent-onset SLE, we reported significant correlations between remission and PROMs [8]. Disease activity (SLE Disease Activity Index-2000) significantly correlated with pain at months 6, 36, and 48 and well-being at month 48 after diagnosis; however, no significant correlation with EQ-5D-3L or fatigue was seen [8]. With concurrent review of our results, different clinical variables may reflect different features of disease burden in SLE. Further studies are required to explore the relationship between clinical disease outcomes and PROMs in patients with SLE.

## Conclusions

These findings illustrate the complexity of SLE disease burden. PROMs in patients with recent-onset SLE, without previous organ damage, remained stable but affected following diagnosis compared with improvement experienced by patients with early RA. Lack of PROMs improvement in patients with SLE may result from disease-related multiorgan system effects, and/or be related to unmet interventional needs that improve health-related QoL. These results highlight the need for improvement in multiprofessional assessment and treatment for patients with SLE.

## Data Availability

The Bristol Myers Squibb policy on data sharing may be found at https://www.bms.com/researchers-and-partners/independent-research/data-sharing-request-process.html.
